# Supplementary material for: A Novel Modeling Optimization Approach for a Seven-Channel Titania Ceramic Membrane in an Oily Wastewater Filtration System Based on Experimentation, Full Factorial Design, and Machine Learning
Source: Membranes (Basel). 2024 Sep 20;14(9):199. doi: 10.3390/membranes14090199 (PMC11433700; doi:10.3390/membranes14090199)
Supplement: Supplementary file 1 [file membranes-14-00199-s001.zip › membranes-3178407-supplementary.pdf]

membranes

Supplementary Material

A Novel Modeling Optimization Approach for a Seven-Channel Titania Ceramic Membrane in an Oily Wastewater Filtration System Based on Experimentation, Full Factorial Design, and Machine Learning

Mohamed Echakouri, Amr Henni * and Amgad Salama

Process Systems Engineering, Produced Water Treatment Laboratory, Faculty of Engineering and Applied Science, University of Regina, Regina, SK S4S 0A2, Canada; echakoum@uregina.ca (M.E.); amgad.salama@uregina.ca (A.S.)
* Correspondence: amr.henni@uregina.ca; Tel.: +1-306-585-4960

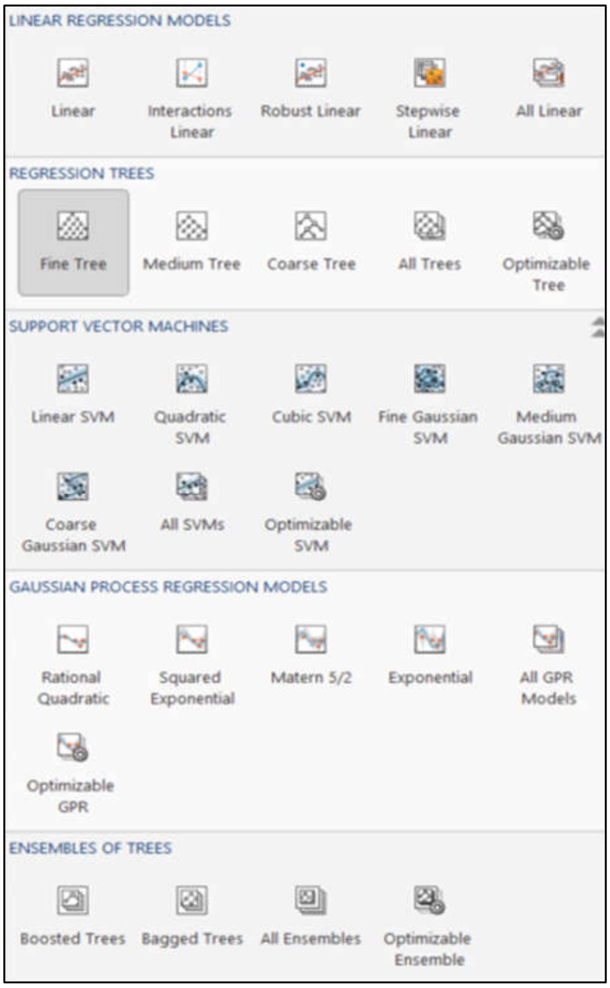

Figure S1. Multiple linear regression models

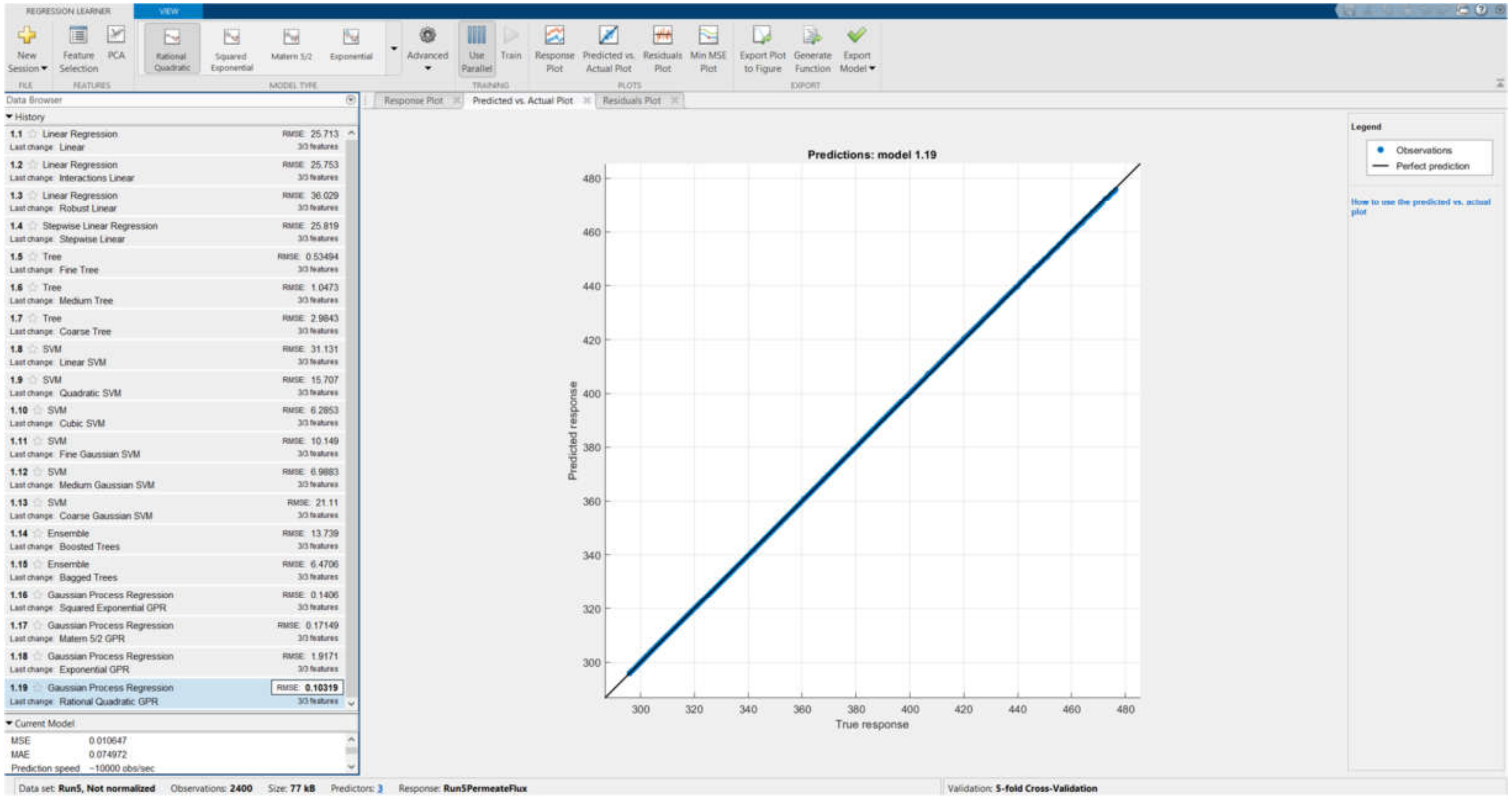

Figure S2. Machine learning flux regression models analysis

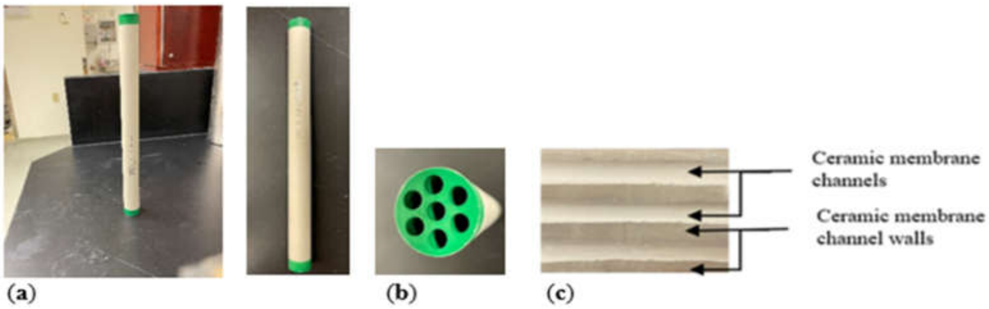

Figure S3. Ceramic membrane dimensions: (**a**) the complete illustration, (**b**) cross-sectional area, (**c**) ceramic membrane internal channels and walls.

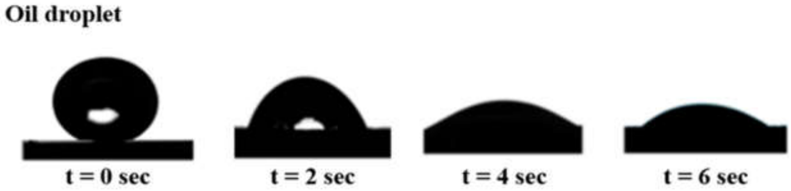

Figure S4. Contact angles of a Bakken oil droplet at the ceramic membrane surface.

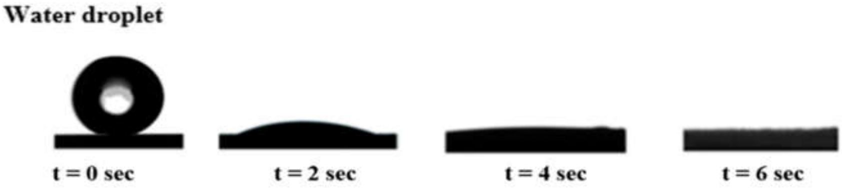

Figure S5. Contact angles of a water droplet at the ceramic membrane surface.

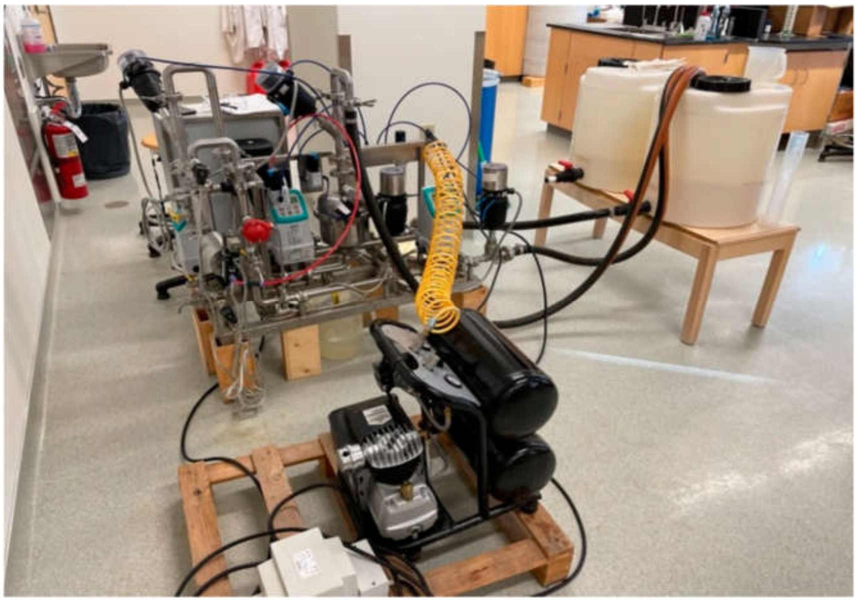

Figure S6. LabBrain crossflow ceramic membrane filtration unit.

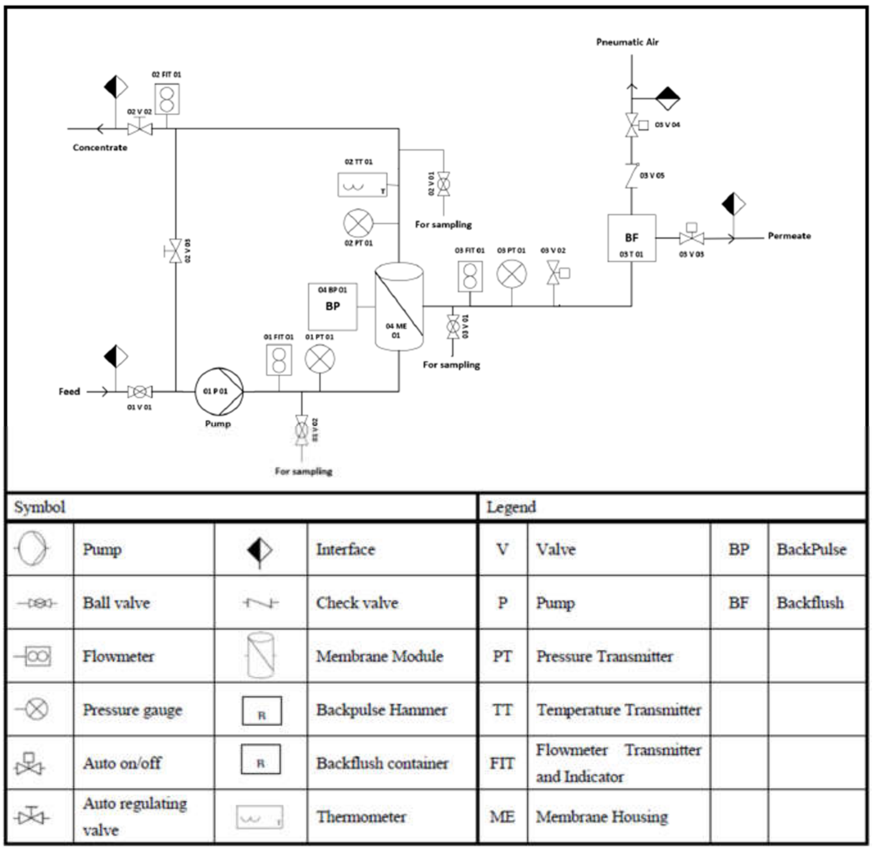

Symbol				Legend			
	Pump		Interface	V	Valve	BP	BackPulse
	Ball valve		Check valve	P	Pump	BF	Backflush
	Flowmeter		Membrane Module	PT	Pressure Transmitter		
	Pressure gauge		Backpulse Hammer	TT	Temperature Transmitter		
	Auto on/off		Backflush container	FIT	Flowmeter Transmitter and Indicator		
	Auto regulating valve		Thermometer	ME	Membrane Housing		

Figure S7. LabBrain P&I *control loop schematic diagram* unit.

	Internal channel ceramic membrane		
	New ceramic membrane (@ t=0 min)	Fouled membrane at the end of the experiment (@ t = 120 min)	Reverse Osmosis Water Membrane Post Cleaning
**Crossflow filtration**	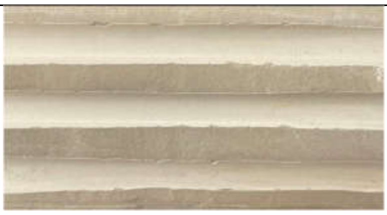	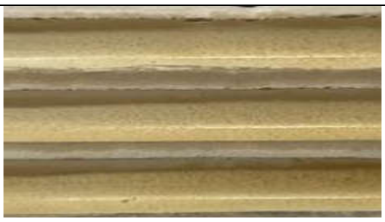	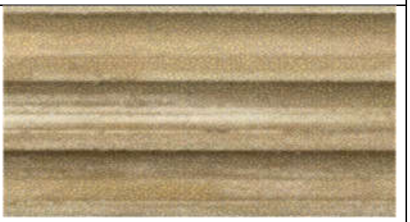

Figure S8. Ceramic membrane cross-sectional view before and after filtration.

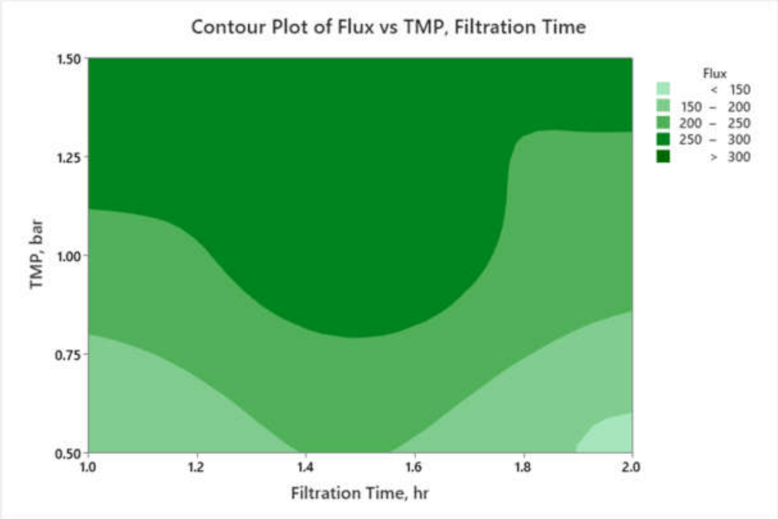

Figure S9. Contour plots for membrane permeate flux as a function of TMP and FT at CFV = 0.75 m/s.

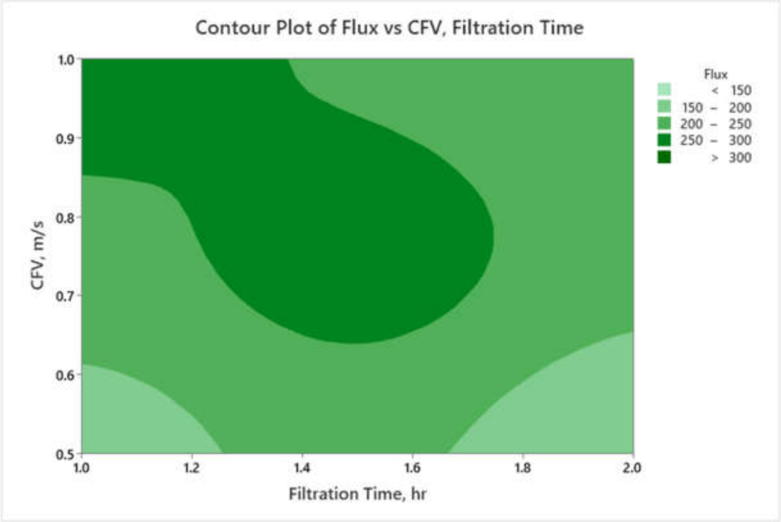

Figure S10. Contour plots for membrane permeate flux as a function of CFV and FT at TMP = 1 bar.

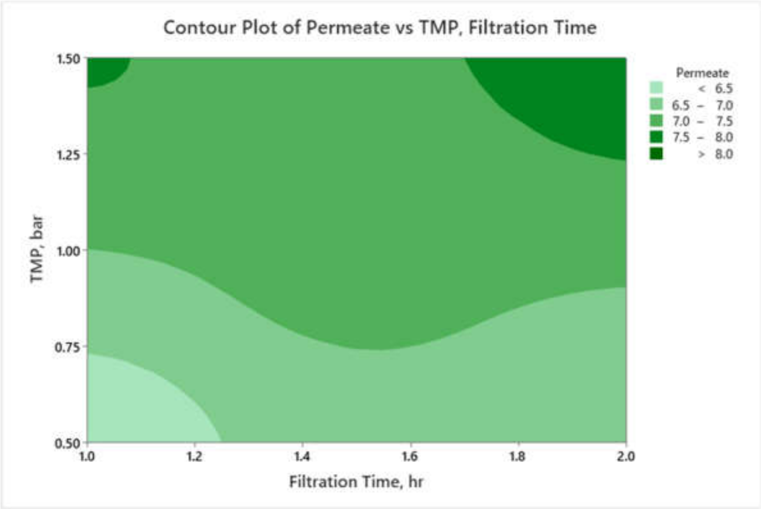

Figure S11. Contour plots for permeate volume as a function of TMP and FT at CFV = 0.75 m/s.

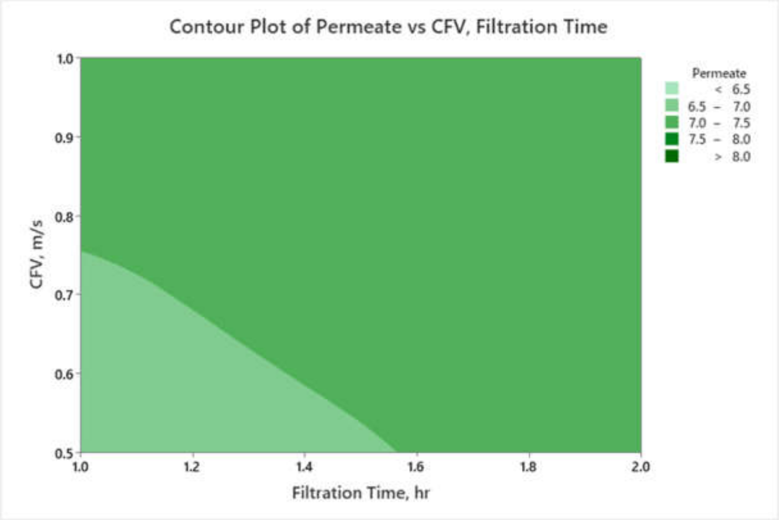

Figure S12. Contour plots for permeate volume as a function of CFV and FT at TMP = 1 bar.

Run 1 (0,0,0)	Run 2 (-1,-1,-1)	Run 3 (1,-1,-1)

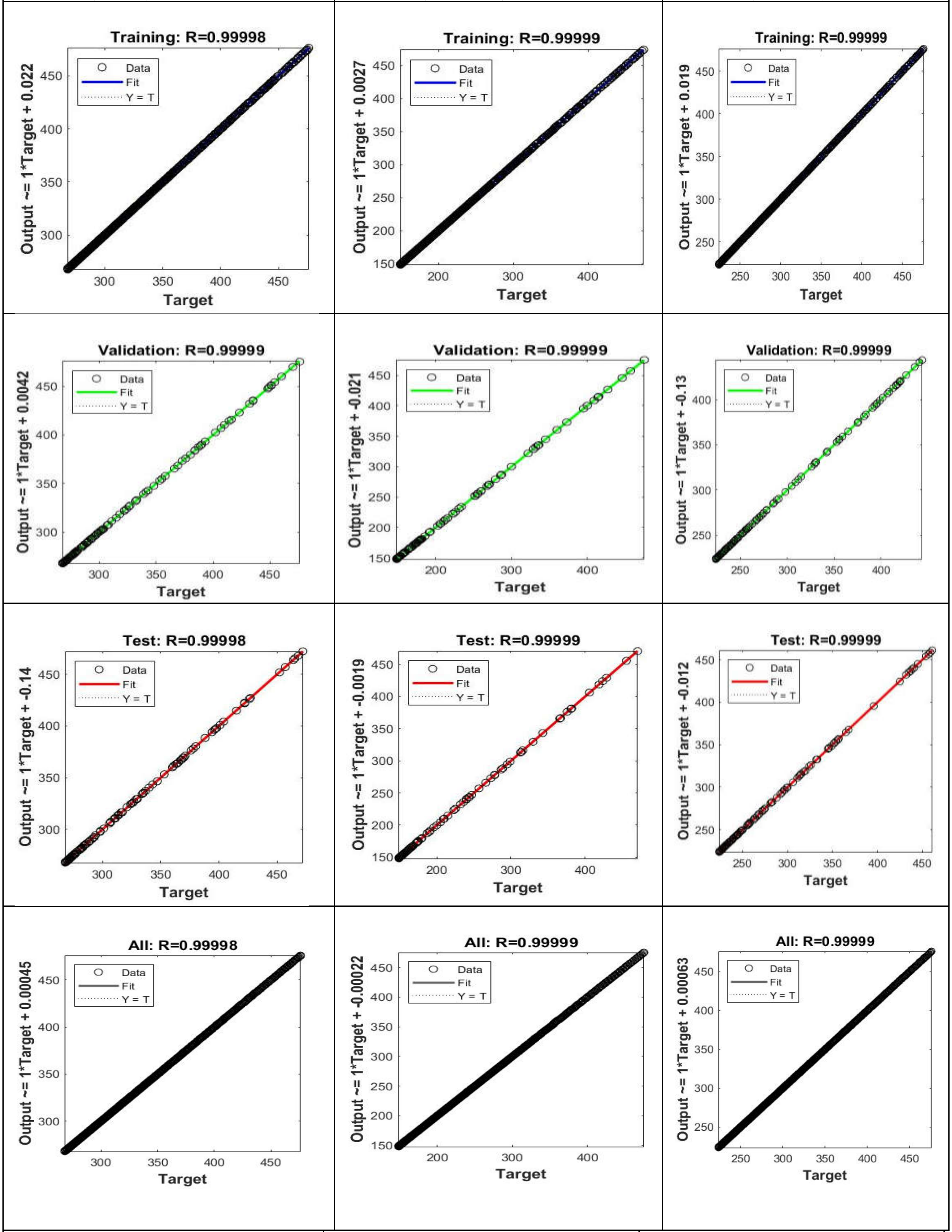

Run 4 (0,0,0)	Run 5 (1,1,1)	Run 6 (1,1,-1)

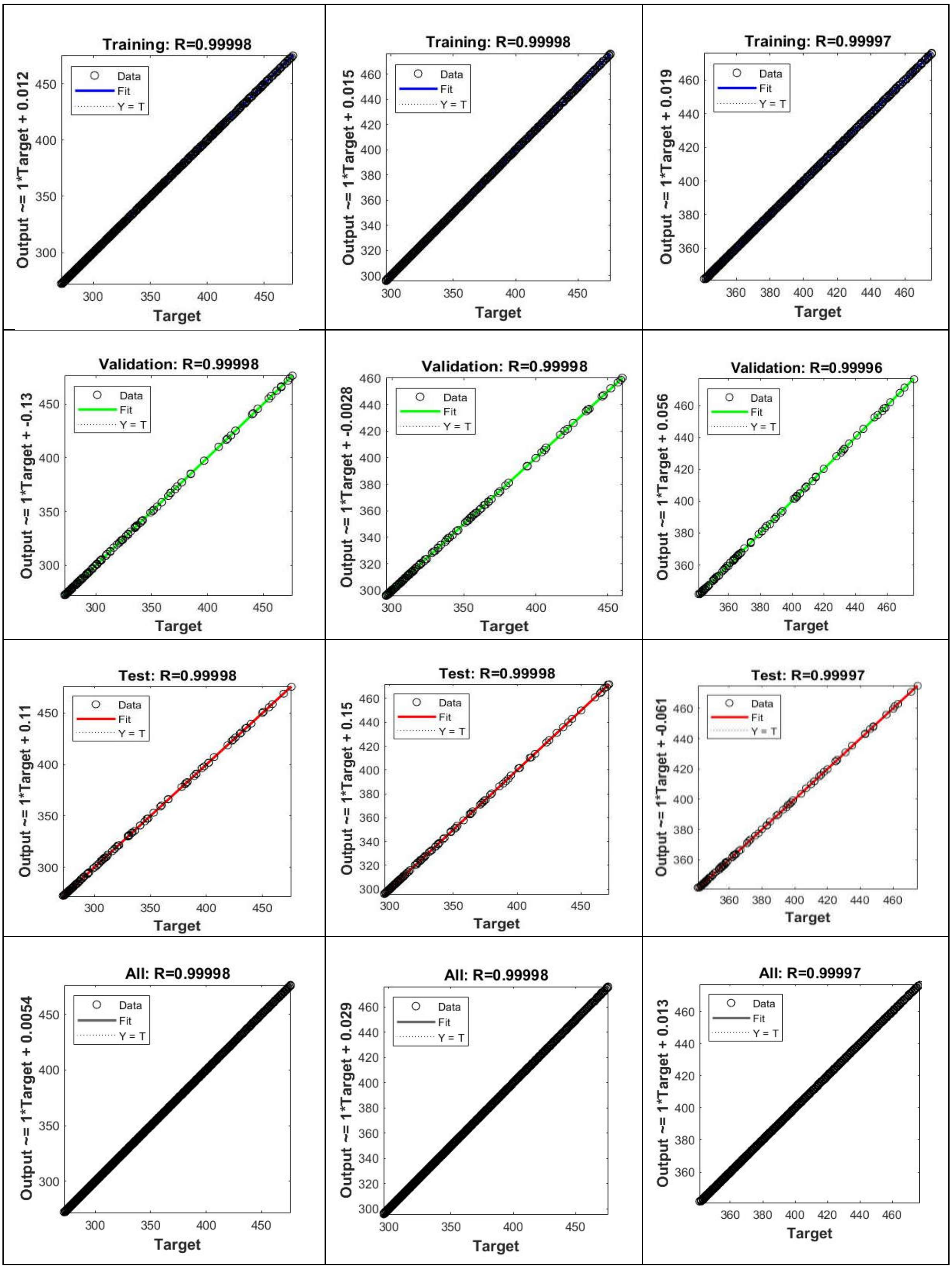
Training: R=0.99998
Output ~= 1*Target + 0.012
Training: R=0.99998
Output ~= 1*Target + 0.015
Training: R=0.99997
Output ~= 1*Target + 0.019
Validation: R=0.99998
Output ~= 1*Target + -0.13
Validation: R=0.99998
Output ~= 1*Target + -0.0028
Validation: R=0.99996
Output ~= 1*Target + 0.056
Test: R=0.99998
Output ~= 1*Target + 0.11
Test: R=0.99998
Output ~= 1*Target + 0.15
Test: R=0.99997
Output ~= 1*Target + -0.061
All: R=0.99998
Output ~= 1*Target + 0.0054
All: R=0.99998
Output ~= 1*Target + 0.029
All: R=0.99997
Output ~= 1*Target + 0.013
Data
Fit
Y = T
Target

Run 7 (1,-1,1)	Run 8 (0,0,0)	Run 9 (-1,1,-1)
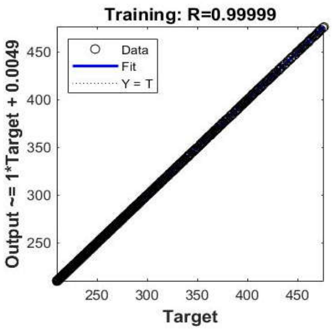	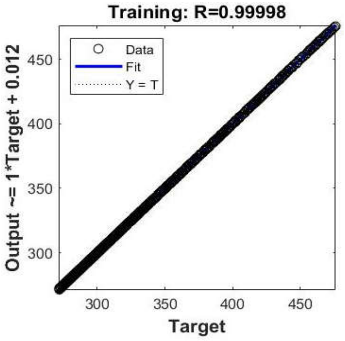	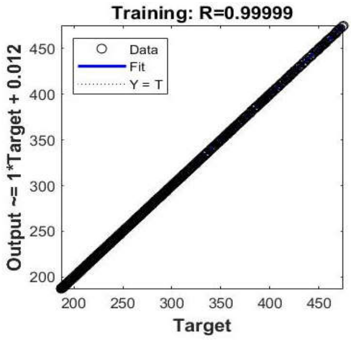
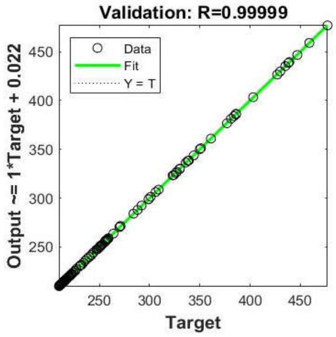	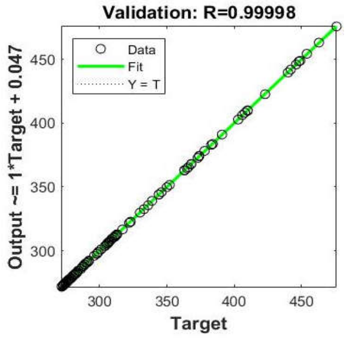	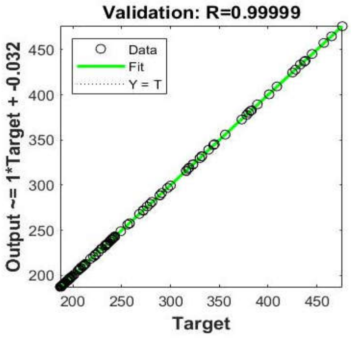
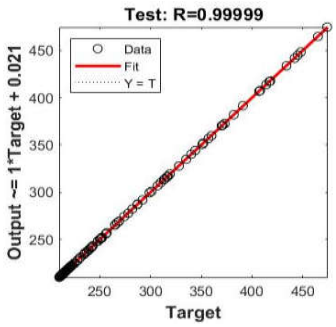	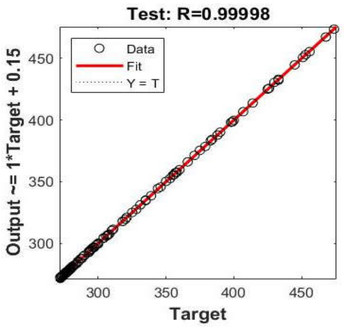	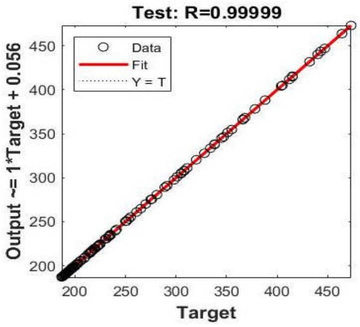
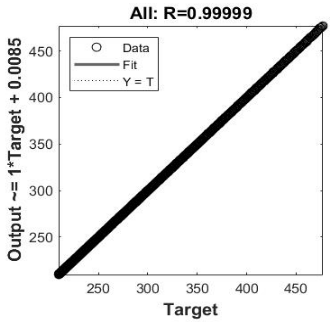	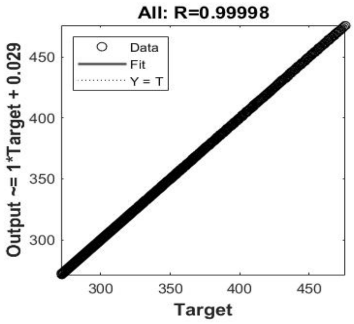	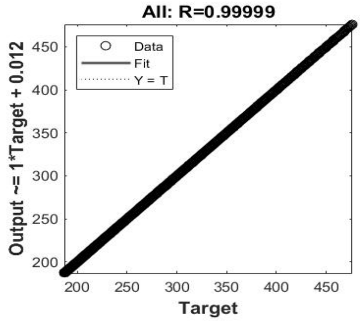

Figure S13. ANN regression plots of the eleven experimental runs

<table>
<tr><th>Run 10 (-1,1,1)</th><th>Run 11 (-1,-1,1)</th></tr>
<tr><td>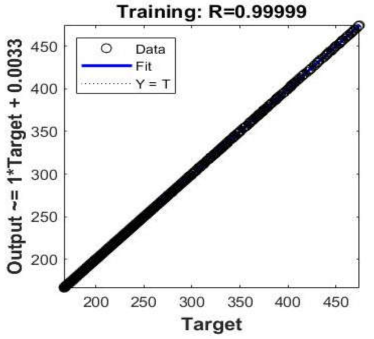
</td><td>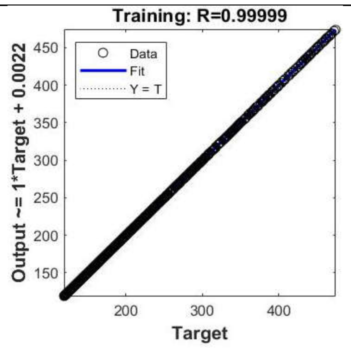
</td></tr>
<tr><td>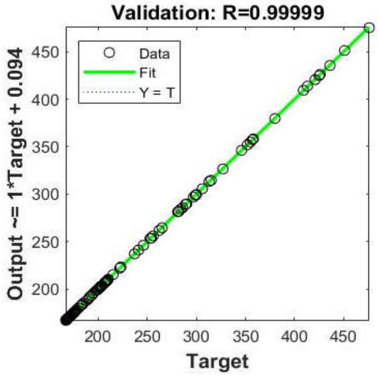
</td><td>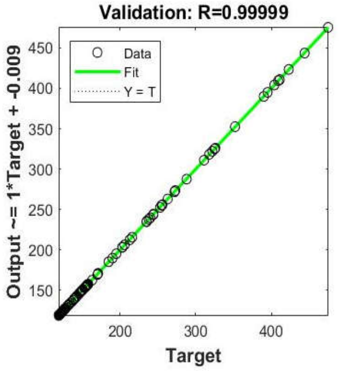
</td></tr>
<tr><td>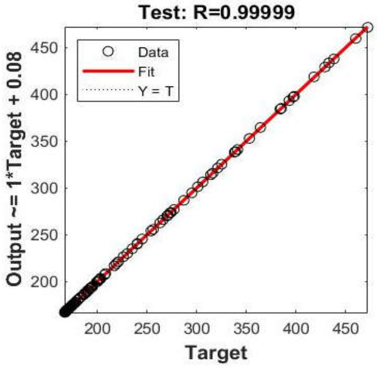
</td><td>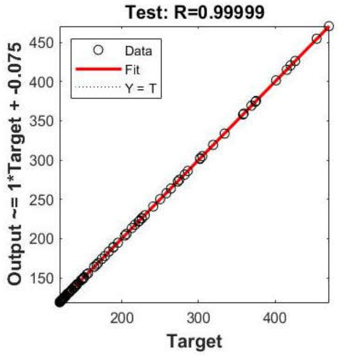
</td></tr>
<tr><td>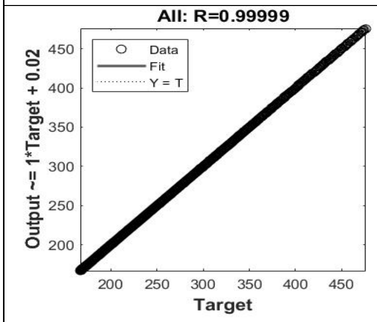
</td><td>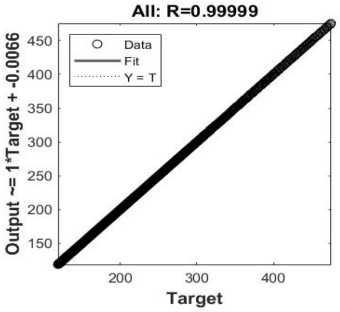
</td></tr>
</table>

All the runs for MLR analysis

Table S1. Algorithms models and their training functions fitted for the experimental run 1

	**Algorithms models**	**Training Function**	**RMSE**	**R^2**	**MSE**	**MAE**	**Training Time, sec**
1	Linear regression	Linear	28.429	0.67	808.2	22.747	6.907
2	Linear regression	Interactions linear	28.501	0.67	812.32	22.788	26.614
3	Linear regression	Robust Linear	35.131	0.49	1234.2	20.255	24.223
4	Stepwise Linear Regression	Stepwise Linear	28.515	0.67	813.12	22.783	23.455
5	Tree	Fine Tree	0.69536	0.99	0.48352	0.40585	22.87
6	Tree	Medium Tree	1.4311	0.99	2.0479	0.73886	21.765
7	Tree	Coarse Tree	3.9243	0.99	15.4	1.9125	21.301
8	SVM	Linear SVM	33.464	0.54	1119.9	20.08	20.574
9	SVM	Quadratic SVM	14.445	0.91	208.66	9.1763	17.922
10	SVM	Cubic SVM	4.484	0.99	20.106	3.3184	17.086
11	SVM	Fine Gaussian SVM	11.473	0.95	131.63	5.4515	16.651
12	SVM	Medium Gaussian SVM	5.9821	0.99	35.785	3.2133	16.014
13	SVM	Coarse Gaussian SVM	20.482	0.83	419.51	10.48	15.245
14	Ensemble	Boosted Trees	13.048	0.93	170.25	12.85	14.343
15	Ensemble	Bagged Trees	8.3093	0.97	69.045	4.7875	13.822
16	Gaussian Process Regression	Squared Exponential GPR	0.11847	0.99	0.014036	0.078545	177.84
17	Gaussian Process Regression	Matern 5/2 GPR	0.22	0.99	0.039999	0.078125	344.26
18	Gaussian Process Regression	Exponential GPR	2.0966	0.99	4.3958	0.66784	172.35
**19**	**Gaussian Regression Process**	**Rational Quadratic GPR**	**0.11581**	**0.99**	**0.013412**	**0.076448**	**632.7**

Table S2. Algorithms models and their training functions fitted for the experimental run 2

	Algorithms models	Training Function	RMSE	R^2	MSE	MAE	Training Time, sec
1	Linear regression	Linear	43.758	0.69	1914.8	35.093	1.2577
2	Linear regression	Interactions linear	43.833	0.69	1921.3	35.102	3.5282
3	Linear regression	Robust Linear	52.554	0.55	2761.9	32.171	3.3376
4	Stepwise Linear Regression	Stepwise Linear	43.396	0.69	1930.4	35.174	3.6711
5	Tree	Fine Tree	1.64412	0.99	2.6936	0.90334	3.04441
6	Tree	Medium Tree	3.4007	0.99	11.565	1.727	2.7862
7	Tree	Coarse Tree	8.2559	0.99	68.161	4.2186	2.5456
8	SVM	Linear SVM	51.327	0.57	2634.5	31.43	3.295
9	SVM	Quadratic SVM	21.204	0.93	449.62	13.653	4.7413
10	SVM	Cubic SVM	0.244	0.99	38.988	4.8416	3.8689
11	SVM	Fine Gaussian SVM	22.668	0.92	513.83	10.961	1.7156
12	SVM	Medium Gaussian SVM	10.514	0.98	110.54	5.3972	1.0433
13	SVM	Coarse Gaussian SVM	33.249	0.82	1105.5	17.154	2.2603
14	Ensemble	Boosted Trees	9.5902	0.99	91.972	8.8499	8.3556
15	Ensemble	Bagged Trees	15.807	0.96	249.87	9.2271	10.743
16	Gaussian Process Regression	Squared Exponential GPR	0.22253	0.99	0.04952	0.16429	117.31
17	Gaussian Process Regression	Matern 5/2 GPR	0.3335	0.99	0.11122	0.14526	188.22
18	Gaussian Process Regression	Exponential GPR	3.7225	0.99	13.857	1.3026	111.69
**19**	**Gaussian Regression Process**	**Rational Quadratic GPR**	**0.20797**	**0.99**	**0.043251**	**0.14847**	**284.6**

Table S3. Algorithms models and their training functions fitted for the experimental run 3

	Algorithms models	Training Function	RMSE	R^2	MSE	MAE	Training Time, sec
1	Linear regression	Linear	29.504	0.80	870.47	24.387	5.8006
2	Linear regression	Interactions linear	29.561	0.79	873.84	24.379	5.5124
3	Linear regression	Robust Linear	30.104	0.79	906.24	23.305	4.8347
4	Stepwise Linear Regression	Stepwise Linear	29.579	0.79	874.94	24.416	8.074
5	Tree	Fine Tree	1.0089	0.99	1.0179	0.66786	7.8541
6	Tree	Medium Tree	2.2571	0.99	5.0945	1.2932	7.5005
7	Tree	Coarse Tree	5.8136	0.99	33.798	3.4576	7.2166
8	SVM	Linear SVM	33.02	0.74	1090.3	22.623	6.8945
9	SVM	Quadratic SVM	10.047	0.98	100.95	7.5186	9.1211
10	SVM	Cubic SVM	4.1641	0.99	17.34	3.7413	8.8379
11	SVM	Fine Gaussian SVM	14.83	0.95	219.92	8.7513	8.5498
12	SVM	Medium Gaussian SVM	5.6381	0.99	31.788	3.6645	8.3157
13	SVM	Coarse Gaussian SVM	16.427	0.94	269.83	9.6486	8.0373
14	Ensemble	Boosted Trees	12.389	0.96	153.49	12.018	9.6828
15	Ensemble	Bagged Trees	12.113	0.97	146.73	7.2512	10.084
16	Gaussian Process Regression	Squared Exponential GPR	0.14564	0.99	0.02121	0.098603	61.78
17	Gaussian Process Regression	Matern 5/2 GPR	0.22425	0.99	0.050287	0.092411	90.139
18	Gaussian Process Regression	Exponential GPR	2.3813	0.99	5.6708	0.78877	62.477
**19**	**Gaussian Regression Process**	**Rational Quadratic GPR**	**0.14351**	**0.99**	**0.020595**	**0.097105**	**123.38**

Table S4. Algorithms models and their training functions fitted for the experimental run 4

	Algorithms models	Training Function	RMSE	R^2	MSE	MAE	Training Time, sec
1	Linear regression	Linear	27.781	0.67	771.8	22.21	4.8178
2	Linear regression	Interactions linear	27.878	0.67	777.18	22.261	7.4377
3	Linear regression	Robust Linear	34.061	0.50	1160.1	19.672	7.1827
4	Stepwise Linear Regression	Stepwise Linear	27.814	0.67	773.64	22.226	8.0331
5	Tree	Fine Tree	0.66027	0.99	0.43595	0..39209	6.9139
6	Tree	Medium Tree	1.2826	0.99	1.645	0.67065	6.6744
7	Tree	Coarse Tree	3.9155	0.99	15.331	1.9036	7.7719
8	SVM	Linear SVM	32.766	0.54	1073.6	19.633	8.3373
9	SVM	Quadratic SVM	14.079	0.91	198.21	8.9187	8.8662
10	SVM	Cubic SVM	4.3986	0.99	19.348	3.2432	10.936
11	SVM	Fine Gaussian SVM	11.359	0.94	129.02	5.3948	9.8362
12	SVM	Medium Gaussian SVM	5.9736	0.98	35.684	3.1058	8.6073
13	SVM	Coarse Gaussian SVM	19.986	0.83	399.45	10.193	9.2706
14	Ensemble	Boosted Trees	13.23	0.92	175.03	13.037	12.266
15	Ensemble	Bagged Trees	8.3314	0.97	69.412	4.7482	13.767
16	Gaussian Process Regression	Squared Exponential GPR	0.11418	0.99	0.013038	0.07809	209.58
17	Gaussian Process Regression	Matern 5/2 GPR	0.19434	0.99	0.037768	0.075552	349.54
18	Gaussian Process Regression	Exponential GPR	2.013	0.99	4.052	0.63274	203.29
**19**	**Gaussian Regression Process**	**Rational Quadratic GPR**	**0.11066**	**0.99**	**0.12246**	**0.075797**	**507.21**

Table S5. Algorithms models and their training functions fitted for the experimental run 5

	Algorithms models	Training Function	RMSE	R^2	MSE	MAE	Training Time, sec
1	Linear regression	Linear	25.713	0.59	661.15	19.97	6.0469
2	Linear regression	Interactions linear	25.753	0.59	663.21	19.963	5.3005
3	Linear regression	Robust Linear	36.029	0.19	1298.1	177.644	9.6615
4	Stepwise Linear Regression	Stepwise Linear	25.819	0.59	666.61	20.016	9.325
5	Tree	Fine Tree	0.53494	0.99	0.28616	0.29344	9.0115
6	Tree	Medium Tree	1.0473	0.99	1.0969	0.49271	8.72998
7	Tree	Coarse Tree	2.9843	0.99	8.9062	1.2966	8.494
8	SVM	Linear SVM	31.131	0.40	969.11	16.998	8.0144
9	SVM	Quadratic SVM	15.707	0.85	246.72	9.1192	7.406
10	SVM	Cubic SVM	6.2853	0.98	39.504	3.8657	10.884
11	SVM	Fine Gaussian SVM	10.149	0.94	103.01	3.9022	10.735
12	SVM	Medium Gaussian SVM	6.9883	0.97	48.836	3.113	10.565
13	SVM	Coarse Gaussian SVM	21.11	0.72	445.64	10.09	10.39
14	Ensemble	Boosted Trees	13.739	0.88	188.76	13.613	11.418
15	Ensemble	Bagged Trees	6.4706	0.97	41.868	3.2969	11.815
16	Gaussian Process Regression	Squared Exponential GPR	0.1406	0.99	0.019768	0.11035	545.17
17	Gaussian Process Regression	Matern 5/2 GPR	00.17149	0.99	0.029408	0.067625	615.09
18	Gaussian Process Regression	Exponential GPR	1.9171	0.99	3.67511	0.54674	313.72
**19**	**Gaussian Regression Process**	**Rational Quadratic GPR**	**0.10319**	**0.99**	**0.010647**	**0.070112**	**113.44**

Table S6. Algorithms models and their training functions ions fitted for the experimental run 6

	**Algorithms models**	**Training Function**	**RMSE**	**R^2**	**MSE**	**MAE**	**Training Time, sec**
1	Linear regression	Linear	15.266	0.81	233.06	12.688	1.1876
2	Linear regression	Interactions linear	15.277	0.81	233.4	12.654	0.95009
3	Linear regression	Robust Linear	15.53	0.81	241.19	12.219	0.82172
4	Stepwise Linear Regression	Stepwise Linear	15.278	0.81	233.43	12.69	2.986
5	Tree	Fine Tree	0.54347	0.99	0.29536	0.36254	0..61165
6	Tree	Medium Tree	1.1196	0.99	1.2535	0.66896	2.8324
7	Tree	Coarse Tree	3.0142	0.99	9.0855	1.8007	2.7174
8	SVM	Linear SVM	16.962	00.77	287.71	11.817	2.6191
9	SVM	Quadratic SVM	4.8753	0.98	23.769	3.7454	2.5074
10	SVM	Cubic SVM	2.2937	0.99	5.2611	2.0552	2.3825
11	SVM	Fine Gaussian SVM	8.5056	0.94	72.346	5.0542	2.2667
12	SVM	Medium Gaussian SVM	2.7675	0.99	7.6591	1.9751	2.1536
13	SVM	Coarse Gaussian SVM	8.0278	0.95	64.445	4.8415	2.0439
14	Ensemble	Boosted Trees	15.969	0.79	25	155.886	4.0774
15	Ensemble	Bagged Trees	6.6427	0.96	44.125	4.292	3.8628
16	Gaussian Process Regression	Squared Exponential GPR	0.062457	0.99	0.0039009	0.04504	52.072
17	Gaussian Process Regression	Matern 5/2 GPR	0.099443	0.99	0.0098889	0.044781	69.53
18	Gaussian Process Regression	Exponential GPR	1.0945	0.99	1.1979	0.39418	48.874
**19**	**Gaussian Regression Process**	**Rational Quadratic GPR**	**0.062188**	**0.99**	**0.0038674**	**0.044647**	**97.315**

Table S7. Algorithms models and their training functions fitted for the experimental run 7

	**Algorithms models**	**Training Function**	**RMSE**	**R^2**	**MSE**	**MAE**	**Training Time, sec**
1	Linear regression	Linear	36.739	0.65	1349.8	29.128	1.8351
2	Linear regression	Interactions linear	36.633	0.65	1342	28.995	1.5596
3	Linear regression	Robust Linear	47.295	0.42	2236.8	25.862	1.3992
4	Stepwise Linear Regression	Stepwise Linear	36.732	0.65	1349.2	299.132	3.0758
5	Tree	Fine Tree	0.94998	0.99	0.90245	0.52139	1.0653
6	Tree	Medium Tree	1.9171	0.99	3.6753	0.91812	0.89322
7	Tree	Coarse Tree	5.0678	0.99	25.683	2.4312	2..897
8	SVM	Linear SVM	43.596	0.50	1900.6	25.573	2.7984
9	SVM	Quadratic SVM	19.843	0.90	393.73	12.194	2.7012
10	SVM	Cubic SVM	6.6846	0.99	44.684	4.6318	3.3783
11	SVM	Fine Gaussian SVM	15.979	0.93	255.33	7.1442	2.5064
12	SVM	Medium Gaussian SVM	8.6115	0.98	74.158	4.3876	2.36
13	SVM	Coarse Gaussian SVM	27.982	0.880	783.01	13.956	2.8723
14	Ensemble	Boosted Trees	11.121	0.97	123.67	10.745	3.0947
15	Ensemble	Bagged Trees	10.075	0.97	101.51	5.663	3.0853
16	Gaussian Process Regression	Squared Exponential GPR	0.15389	0.99	0.023682	0.11191	159.26
17	Gaussian Process Regression	Matern 5/2 GPR	0.2544	0.99	0.064719	0.10792	266.67
18	Gaussian Process Regression	Exponential GPR	2.749	0.99	7.5571	0.90354	145.66
**19**	**Gaussian Regression Process**	**Rational Quadratic GPR**	**0.14963**	**0.99**	**0.02239**	**0.10718**	**400.54**

Table S8. Algorithms models and their training functions fitted for the experimental run 8

	Algorithms models	Training Function	RMSE	R^2	MSE	MAE	Training Time, sec
1	Linear regression	Linear	27.758	0.67	770.49	22.207	4.3776
2	Linear regression	Interactions linear	27.768	0.67	771.08	22.206	8.6775
3	Linear regression	Robust Linear	33.922	0.51	1150.7	19.656	8.3485
4	Stepwise Linear Regression	Stepwise Linear	27.814	0.67	773.6	22.226	8.0916
5	Tree	Fine Tree	0.65681	0.99	0.4314	0.39461	7.8005
6	Tree	Medium Tree	1.2283	0.99	1.5088	0.65118	7.3483
7	Tree	Coarse Tree	3.9777	0.99	15.822	1.9508	6.8737
8	SVM	Linear SVM	32.941	0.53	1085.1	19.615	11.456
9	SVM	Quadratic SVM	14.158	0.91	200.44	8.9564	11.137
10	SVM	Cubic SVM	4.3763	0.99	19.152	3.2418	10.799
11	SVM	Fine Gaussian SVM	11.314	0.95	128	5.3063	10.478
12	SVM	Medium Gaussian SVM	6.1635	0..98	37.988	3.1359	9.8724
13	SVM	Coarse Gaussian SVM	20.03	0.83	401.18	10.223	9.4104
14	Ensemble	Boosted Trees	13.207	0.93	174.43	13.019	12.467
15	Ensemble	Bagged Trees	8.2759	0.97	68.491	4.6454	11.834
16	Gaussian Process Regression	Squared Exponential GPR	0.11037	0.99	0.012181	0.077144	168.74
17	Gaussian Process Regression	Matern 5/2 GPR	0.18257	0.99	0.03333	0.071992	280.2
18	Gaussian Process Regression	Exponential GPR	1.9958	0.99	3.9832	0.65374	155.7
**19**	**Gaussian Regression Process**	**Rational Quadratic GPR**	**0.10843**	**0.99**	**0.011757**	**0.074881**	**399.61**

Table S9. Algorithms models and their training functions fitted for the experimental run 9

	Algorithms models	Training Function	RMSE	R^2	MSE	MAE	Training Time, sec
1	Linear regression	Linear	32.667	0.81	1067.1	27.128	4.7646
2	Linear regression	Interactions linear	32.708	0..81	1069.88	27.13	4.5421
3	Linear regression	Robust Linear	33.311	0.80	1109.7	26.143	4.3286
4	Stepwise Linear Regression	Stepwise Linear	32.661	0.81	1066.8	27.117	6.7763
5	Tree	Fine Tree	1.1321	0..99	1.2817	0.75576	6.5779
6	Tree	Medium Tree	2.3012	0.99	5.2956	1.4001	6.3933
7	Tree	Coarse Tree	6.7389	0.99	45.412	4.0861	6.1694
8	SVM	Linear SVM	36.259	0.77	1314.7	25.292	77.8145
9	SVM	Quadratic SVM	10.523	0.98	110.73	8.006	7.6279
10	SVM	Cubic SVM	4.7712	0.99	22.764	4.2537	7.4558
11	SVM	Fine Gaussian SVM	16.095	0.95	259.04	9.9045	8.2249
12	SVM	Medium Gaussian SVM	5.9298	0.99	35.163	4.2364	7.1841
13	SVM	Coarse Gaussian SVM	17.177	0.95	295.05	10.399	6.9416
14	Ensemble	Boosted Trees	11.495	0.98	132.13	10.952	9.7087
15	Ensemble	Bagged Trees	15.197	0.96	230.95	10.06	10.186
16	Gaussian Process Regression	Squared Exponential GPR	0.14689	0.99	0.021576	0.10004	57.39
17	Gaussian Process Regression	Matern 5/2 GPR	0.22347	0.99	0.0499937	0.096519	66.298
18	Gaussian Process Regression	Exponential GPR	2.4836	0.99	6.1681	0.85892	62.774
**19**	**Gaussian Regression Process**	**Rational Quadratic GPR**	**0.14578**	**0.99**	**0.021251**	**0.099163**	**132.73**

Table S10. Algorithms models and their training functions fitted for the experimental run 10

	Algorithms models	Training Function	RMSE	R^2	MSE	MAE	Training Time, sec
1	Linear regression	Linear	44.796	0.48	2006.6	33.015	4.6327
2	Linear regression	Interactions linear	44.908	0.48	2016.7	33.063	4.4272
3	Linear regression	Robust Linear	65.591	0.11	4302.2	28.354	4.2213
4	Stepwise Linear Regression	Stepwise Linear	44.813	0.48	2008.2	33.026	6.5623
5	Tree	Fine Tree	0.91438	0.99	0.8361	0.42329	6.3459
6	Tree	Medium Tree	2.1937	0.99	4.8125	0.83907	6.1669
7	Tree	Coarse Tree	5.2123	0.99	27.168	2.0812	5.9854
8	SVM	Linear SVM	55.789	0.20	3112.4	26.17	6.8465
9	SVM	Quadratic SVM	34.474	0.69	1188.5	17.14	7.1263
10	SVM	Cubic SVM	18.124	0.92	328.48	9.0185	8.0766
11	SVM	Fine Gaussian SVM	25.573	0.83	654	8.0496	8.572
12	SVM	Medium Gaussian SVM	21.217	0.88	450.16	7.9039	7.88
13	SVM	Coarse Gaussian SVM	44.342	0.49	1966.2	18.84	8.3659
14	Ensemble	Boosted Trees	8.9627	0.98	80.33	8.4859	10.992
15	Ensemble	Bagged Trees	10.478	0.97	109.79	5.0743	10.811
16	Gaussian Process Regression	Squared Exponential GPR	0.26835	0.99	0.072011	0.18279	586.1
17	Gaussian Process Regression	Matern 5/2 GPR	0.5149	0.99	0.26512	0.18982	488.06
18	Gaussian Process Regression	Exponential GPR	4.502	0.99	20.268	1.3263	268.85
**19**	**Gaussian Regression Process**	**Rational Quadratic GPR**	**0.16317**	**0.99**	**0.063572**	**0.27709**	**474.03**

Table S11. Algorithms models and their training functions fitted for the experimental run 11

	Algorithms models	Training Function	RMSE	R^2	MSE	MAE	Training Time, sec
1	Linear regression	Linear	51.235	0.44	2625	36.86	3.7994
2	Linear regression	Interactions linear	51.311	0.44	2632.8	36.819	3.5066
3	Linear regression	Robust Linear	73.471	0.15	5398	30.335	5.5203
4	Stepwise Linear Regression	Stepwise Linear	51.24	0.44	2625.6	36.841	5.3291
5	Tree	Fine Tree	1.2376	0.99	1.5316	0.52774	5.1203
6	Tree	Medium Tree	2.5712	0.99	6.6113	0.96842	4.9658
7	Tree	Coarse Tree	6.0438	0.99	36.528	2.368	4.803
8	SVM	Linear SVM	64.328	0.12	4138.1	28.14	5.6889
9	SVM	Quadratic SVM	42.652	0.61	1819.2	19.829	5.4898
10	SVM	Cubic SVM	24.698	0.87	610	11.473	4.0662
11	SVM	Fine Gaussian SVM	35.43	0.73	1255.3	14.495	4.915
12	SVM	Medium Gaussian SVM	30.177	0.81	910.66	10.508	3.8976
13	SVM	Coarse Gaussian SVM	53.824	0.38	2897	21.698	4.6595
14	Ensemble	Boosted Trees	7.2421	0.99	52.448	6.5008	7.3543
15	Ensemble	Bagged Trees	12.016	0.97	144.39	5.3114	7.6324
16	Gaussian Process Regression	Squared Exponential GPR	0.26505	0.99	0.070251	0.16968	697.1
17	Gaussian Process Regression	Matern 5/2 GPR	0.65118	0.99	0.42404	0.28285	557.8
18	Gaussian Process Regression	Exponential GPR	5.3675	0.99	28.81	1.6417	300.29
**19**	**Gaussian Regression Process**	**Rational Quadratic GPR**	**0.23217**	**0.99**	**0.31526**	**1.15277**	**271.19**
